# Effect of Acid Flow Rate, Membrane Surface Area, and Capture Solution on the Effectiveness of Suspended GPM Systems to Recover Ammonia

**DOI:** 10.3390/membranes11070538

**Published:** 2021-07-16

**Authors:** María Soto-Herranz, Mercedes Sánchez-Báscones, Juan Manuel Antolín-Rodríguez, Matías B. Vanotti, Pablo Martín-Ramos

**Affiliations:** 1 Department of Agroforestry Sciences, ETSIIAA, University of Valladolid, Avenida de Madrid 44, 34004 Palencia, Spain; msanchez@agro.uva.es (M.S.-B.); juanmanuel.antolin@uva.es (J.M.A.-R.); 2 United States Department of Agriculture (USDA), Agricultural Research Service, Coastal Plains Soil, Water and Plant Research Center, 2611W. Lucas St., Florence, SC 29501, USA; Matias.Vanotti@ars.usda.gov; 3 Instituto Universitario de Investigación en Ciencias Ambientales de Aragón (IUCA), EPS, Universidad de Zaragoza, Carretera de Cuarte, s/n, 22071 Huesca, Spain; pmr@unizar.es

**Keywords:** acid flow rate, ammonia capture solution, gas-permeable membrane, mass flow, surface area, suspended system

## Abstract

Ammonia losses from manure pose serious problems for ecosystems and human and animal health. Gas-permeable membranes (GPMs) constitute a promising approach to address the challenge of reducing farm ammonia emissions and to attain the EU’s Clean Air Package goals. In this study, the effect of NH_3_-N concentration, membrane surface area, acid flux, and type of capture solution on ammonia recovery was investigated for a suspended GPM system through three experiments, in which ammonia was released from a synthetic solution (NH_4_Cl + NaHCO_3_ + allylthiourea). The effect of two surface areas (81.7 and 163.4 cm^2^) was first evaluated using three different synthetic N emitting concentrations (3000, 6000, and 12,000 mg NH_3_-N∙L^−1^) and keeping the flow of acidic solution (1N H_2_SO_4_) constant (0.8 L·h^−1^). A direct relationship was found between the amount of NH_3_ captured and the NH_3_-N concentration in the N-emitting solution, and between the amount of NH_3_ captured and the membrane surface area at the two lowest concentrations. Nonetheless, the use of a larger membrane surface barely improved ammonia capture at the highest concentration, pointing to the existence of other limiting factors. Hence, ammonia capture was then studied using different acid flow rates (0.8, 1.3, 1.6, and 2.1 L∙h^−1^) at a fixed N emitting concentration of 6000 mg NH_3_-N∙L^−1^ and a surface area of 122.5 cm^2^. A higher acid flow rate (0.8–2.1 L∙h^−1^) resulted in a substantial increase in ammonia absorption, from 165 to 262 mg of NH_3_∙d^−1^ over a 14-day period. Taking the parameters that led to the best results in experiments 1 and 2, different types of ammonia capture solutions (H_2_SO_4_, water and carbonated water) were finally compared under refrigeration conditions (at 2 °C). A high NH_3_ recovery (81% in 7 days), comparable to that obtained with the H_2_SO_4_ solution (88%), was attained when chilled water was used as the capture solution. The presented results point to the need to carefully optimize the emitter concentration, flow rate, and type of capture solution to maximize the effectiveness of suspended GPM systems, and suggest that chilled water may be used as an alternative to conventional acidic solutions, with associated savings.

## 1. Introduction

Nitrogen is a key nutrient widely used in agricultural fertilization, since its most available forms in the soil are generally insufficient to satisfy crop requirements, and it is also the majority element in manure [1], which has been historically used as a soil amendment. However, part of the nitrogen contained in manure is lost to the atmosphere in the form of ammonia, resulting in a significant reduction of its fertilizer value [2], and contributing to the formation of acid rain, acid deposition, and eutrophication [3]. It also contributes to the formation of fine particulate matter (PM 2.5), considered a major environmental risk to human health [4]. Consequently, Directive (EU) 2016/2284 has established emission ceilings for ammonia emissions and has laid down national emission reduction commitments. In the particular case of Spain, which exceeds the ammonia emission ceilings, a reduction of NH_3_ emissions by 16% by 2030 is required.

The aforementioned emission of NH_3_ from manure depends on a variety of factors, including temperature, ventilation, humidity, density of animals, soil conditions, and feed composition. The NH_3_ volatilization process involves its movement to the manure surface by diffusion and its subsequent release into the air, mainly through convective mass transfer [5,6]. In general, NH_3_ volatilization increases as a function of the NH_4_^+^/NH_3_ concentration in manure, the wind speed, the turbulence on the manure surface, as well as the temperature and the acidity of the manure [6,7,8,9].

In recent years, different approaches have been developed to mitigate NH_3_ emissions. Improvements have been made in ventilation and emission capture systems, types of accommodation, and manure storage management [10,11,12], while diverse measures have been taken in terms of the management and treatment of manure, such as acidification, solid–liquid separation, or the use of covers in slurry ponds [13,14,15]. As regards new technologies for NH_3_ emission abatement in livestock operations, they are being focused on N recovery. These technologies include reverse osmosis [16], air-stripping using stripping towers and acid absorption [17], zeolite adsorption through ion exchange [18], co-precipitation with phosphate and magnesium to form struvites [19], use of bio-adsorbents, and gas-permeable membranes (GPM) [20]. Traditional processes suffer from some limitations: reverse osmosis requires high pressure; air stripping towers and zeolite adsorption techniques require manure pre-treatment; precipitation of struvites not only requires the use of additives but may also interfere with equipment performance and lead to increased maintenance costs [20]; and research is still lacking on the reusability of ammonium-loaded bio-adsorbents as bio-fertilizers or even bio-compost [21,22]. On the other hand, GPM technology has a low energy consumption (0.18 kWh·kg NH_3_^−1^), requires a small working pressure, does not require pre-treatment of effluents, does not need the addition of any alkaline reagent [23,24], and does not drastically disturb the operation of the livestock activity, which can all be regarded as interesting advantages.

Numerous studies on the recovery of total ammoniacal nitrogen (TAN) in different types of polluting sources such as chicken manure, pig manure, anaerobically digested slurry, radioactive wastewater, or digested chicken manure, have shown that the GPM technique is very effective for the recovery of NH_3_, reducing the concentration of TAN in the emission sources in a short period of time [7,25,26,27,28,29,30]. Moreover, this method can be used both to remove NH_3_ from liquid manure before it escapes into the air [20,25] and to recover volatilized NH_3_ directly from the air [27,28].

Gas-permeable hydrophobic membranes can be made of polyethylene (PE), polypropylene (PP), polyvinyl chloride (PVC), polyvinylidene fluoride (PVDF), fluorinated ethylene propylene (FEP), perfluoroalkoxy (PFA), ethylenetetrafluoroethylene (eTFE), polyetheretherketone (PEEK), polytetrafluoroethylene (PTFE), and expanded polytetrafluoroethylene (ePTFE). This latter material—which is microporous, flexible, and hydrophobic—is particularly interesting due to its high permeability rate for low-pressure gas flow differentials between the inside and outside of the ePTFE tube.

Regardless of the material used, the gas separation process involves the flow of NH_3_ through the hydrophobic microporous membrane by diffusion, followed by ammonia capture in a receptor solution that circulates inside the membrane. NH_3_ then combines with the free protons (H^+^) of the acid to form non-volatile ammonia (NH_4_^+^). The flow rate and the concentration of free ammonia (FA) in the manure are the two main parameters that influence this process [20,31,32,33,34]. There are also some studies on the behavior of the permeate on the rate of ammonia diffusion using membrane contactors [35,36]. In contrast, the influence of the chemistry of certain capture solutions on the absorption of ammonia using ePTFE membranes has not yet been addressed.

While there are numerous studies in which different operational parameters have been evaluated with hydrophobic ePTFE membranes immersed in the emitting source of N, the available information on the behavior of ePTFE membranes suspended in an air system is limited. Therefore, the aim of this study was to evaluate the influence of parameters such as the acid flow rate, membrane surface area, NH_3_ concentration in the N-emitting solution, and type of capture solution on the NH_3_ capture efficiency using hydrophobic ePTFE membranes in a suspended system inside a hermetic chamber. The results of these laboratory assays were used to optimize design parameters of a pilot-scale prototype for cleaning the air of animal housing, in the framework of the LIFE+ Ammonia Trapping (LIFE15-ENV/ES/000284) project.

## 2. Materials and Methods

### 2.1. Experimental Design

**Figure 1:**
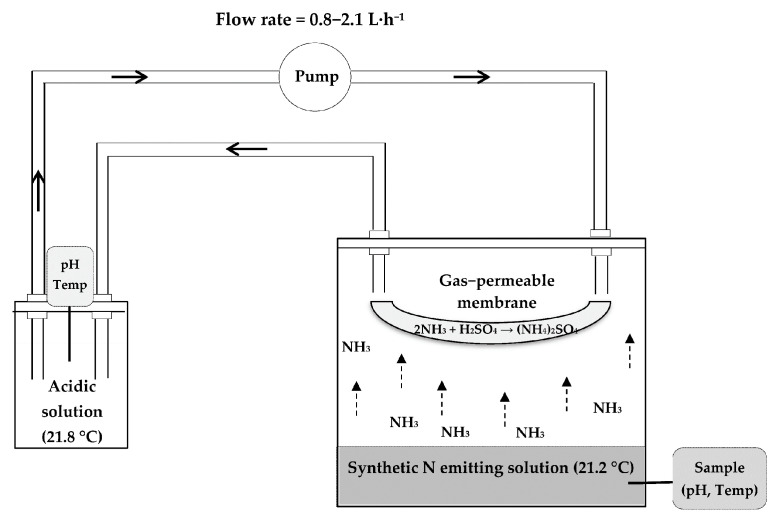
Diagram of the NH_3_ capture process by the suspended gas permeable membrane system in a closed circuit.

The experimental design (Figure 1) consisted of two 11-L hermetic chambers, into which a N emitting solution was poured to recover the NH_3_ gas emitted through a gas permeable membrane, using the method developed by Szogi, et al. [37].

Synthetic N emitting solutions were used in order to minimize the variability caused by the use of manures with inconstant ammonia concentrations. An amount of 1 L of synthetic solution was placed inside of each chamber, with the following composition: 11.9 g NH_4_Cl·L^−1^ + 20.9 g NaHCO_3_·L^−1^ (3000 mg NH_3_-N·L^−1^); 23.8 g NH_4_Cl·L^−1^ + 41.8 g NaHCO_3_·L^−1^ (6000 mg NH_3_-N·L^−1^); and 47.5 g NH_4_Cl·L^−1^ and 83.6 g NaHCO_3_·L^−1^ (12,000 mg NH_3_-N·L^−1^). In all of them, 10 mg∙L^−1^ of allythiourea (98%) was added as a nitrification inhibitor, according to the procedure reported in other assays [38].

The acidic solution reservoir used to capture the ammonia contained 1 L of N capturing solution (1N H_2_SO_4_). This solution was continuously recirculated inside the membrane using a peristaltic pump (Pumpdrive 5001, Heidolph, Schwabach, Germany).

Gas-permeable tubing made of ePTFE (Zeus Industrial Products Inc., Orangeburg, SC, USA) was used for NH_3_ capture, with an outer diameter of 5.2 mm, a wall thickness of 0.64 mm, a polymer density of 0.95 g·cm^−3^, a porosity < 60%, average pore size length of 12.7 ± 5.9 µm and average pore size width of 1.3 ± 0.9 µm. The pores of the ePTFE membrane were elongated in the extrusion process. As shown in Figure 1, the GPM was suspended in the chamber, not immersed in the emitting source.

Three experiments were conducted: in the first experiment, the influence of the membrane surface area on NH_3_ capture was evaluated for three NH_3_ concentrations in the synthetic N emitting solution (viz. 3000, 6000, and 12,000 mg NH_3_-N·L^−1^). Two different membrane surfaces (81.7 cm^2^ and 163.4 cm^2^) were assayed, keeping a constant flow rate (0.8 L∙h^−1^).

In the second experiment, the effect of the acid flow rate on NH_3_ recovery was evaluated. An intermediate membrane area surface (122.5 cm^2^) and NH_3_ concentration in the synthetic N emitting solution (6000 mg NH_3_-N L^−1^) were chosen, testing four different acid flow rates (viz. 0.8, 1.3, 1.6, and 2.1 L·h^−1^).

**Figure 2:**
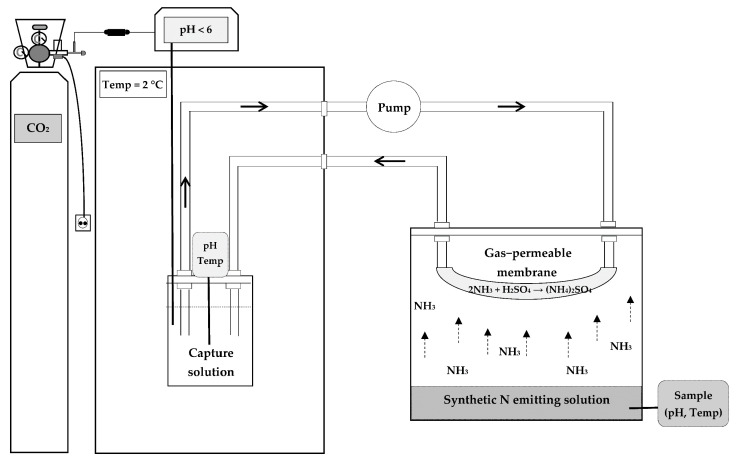
Diagram of the NH_3_ capture process with different ammonia capture solutions.

In the third experiment, the impact of the type of capture solution on the recovery of NH_3_ was analyzed. To do so, the parameters that led to the best results in the two previous experiments were selected (viz., a membrane surface area of 163.4 cm^2^, a NH_3_ concentration in the synthetic N emitting solution of 6000 mg of NH_3_-N·L^−1^, and a flow rate of 2.1 L·h^−1^), and three types of ammonia capture solution were assayed: 1N H_2_SO_4_, deionized water, and carbonated water. For the latter, CO_2_ was alternately dosed in the carbonated water traps at a pressure of 0.1 bar as a function of the pH present in the medium (pH < 6.36). The traps remained under constant refrigeration at 2 °C in order to increase the solubility capacity of CO_2_ [39,40]. A diagram of the NH_3_ capture process with different capture solutions is shown in Figure 2.

It should be clarified that the different surface areas used in the aforementioned tests were obtained by cutting different lengths of material (50, 75, and 100 cm for 81.7, 122.5, and 163.4 cm^2^, respectively). In experiments #1 and #2, three replicates were performed for each assay over a period of 14 days. In experiment 3, the three replicates were carried out over a 7-day period.

### 2.2. Analysis Methodology

The pH, temperature, and NH_3_-N concentration were monitored both in the capture solutions and in the synthetic N emitting solutions. The pH of the acidic solution remained below 2 and that of the synthetic solution above 8, in agreement with Garcia-González and Vanotti [20]. In the third experiment with deionized water, the pH was kept below 8.1 [41]; and, in the case of carbonated water, it was kept below 6.36 to favor the predominance of the H_2_CO_3_ form in the medium to react with ammonia [42].

The pH and temperature were measured with a Crison GLP22 m (Crison Instruments S.A., Barcelona, Spain). NH_3_-N concentration was determined by distillation (with a Kjeltec^TM^ 8100 nitrogen distillation unit; Foss Iberia S.A., Barcelona, Spain), through capture of distillate in borate buffer and subsequent titration with 0.2 mol·L^−1^ HCl [43]. To measure NH_3_ gas concentration inside the chamber, the gas was collected using a colorimetric tube (Gastec 3La; 3M, Japan; error range: ±5%).

### 2.3. Data Calculations

The NH_3_-N mass removed (expressed in mg NH_3_-N) was determined as the difference between the amount of NH_3_-N at the beginning (initial NH_3_-N) and at the end of the experiment in the synthetic N emitting solution. The NH_3_-N mass recovered (mg NH_3_-N) was determined by the amount of NH_3_-N captured at the end of the experiment in the acidic solution. The N removal efficiency (%) was estimated by dividing the recovered mass by the removed mass.

The NH_3_-N mass flow or N flux through the membrane (*J*, expressed in mg NH_3_-N·cm^−2^·d^−1^), which occurs as a consequence of the gas concentration gradient across the membrane [32,44], was determined by considering the N mass captured per day and the surface area of the GPM tubing.

### 2.4. Statistical Analyses

The results were analyzed using one-way analysis of variance (ANOVA), followed by post hoc comparison of means through Tukey’s test at *p* < 0.05. R statistical software was used for the statistical analyses [45].

## 3. Results and Discussion

### 3.1. Effect of Membrane Surface Area

As noted above, two membrane surfaces areas (81.7 and 163.4 cm^2^) and three concentrations of synthetic N emitting solutions (3000, 6000, and 12,000 mg NH_3_-N·L^−1^) were tested, keeping the acidic solution flow rate fixed at 0.8 L∙h^−1^. The pH value in the synthetic N emitting solution was 8.3 ± 0.1, and the temperature was 21.5 ± 0.4 °C.

The NH_3_-N mass removed from the synthetic N emitting solution and recovered by the acidic solution, and the N flux for each combination of membrane surface area and N concentration in the synthetic solution are summarized in Table 1.

The ammonia emission percentages were 30.6, 29.5, and 43.4%, and 53.4, 50.4, and 42.2% for the 3000, 6000, and 12,000 mg NH_3_-N·L^−1^ concentrations and the 81.7 and 163.4 cm^2^ membrane surface areas, respectively.

The NH_3_ recovery percentage was higher than 70% in all cases. However, significant differences were observed as a function of the membrane surface area and NH_3_-N concentration in the synthetic solution, with the highest recovery percentage (88.1%) for the combination of the largest membrane surface area (163.4 cm^2^) and the highest NH_3_-N concentration in the synthetic solution (12,000 mg NH_3_-N·L^−1^).

For the same membrane area, the recovered NH_3_-N increased proportionally to the ammonia content in the synthetic solution, in good agreement with Fillingham, et al. [46], who observed a linear increase in the recovered NH_3_ capture rate as the concentration of TAN in NH_4_Cl solutions increased from 1000 to 3600 mg NH_3_-N·L^−1^, and with Sürmeli, et al. [7], who obtained a 12% higher recovery of ammonia with PDMS membranes in more concentrated digestates (4000 mg·L^−1^) compared to less concentrated ones (3000 mg·L^−1^). However, while at the 3000 and 6000 mg NH_3_-N·L^−1^ concentrations the amount of recovered NH_3_ approximately doubled in line with the membrane surface area, such direct relationship was not observed at the highest concentration of 12,000 mg NH_3_-N·L^−1^, with a mere 10% increase when the membrane surface area doubled. This suggests that another limiting factor, such as the flow rate, should be taken into consideration.

With regard to the N flux, this increased as the concentration of NH_3_-N increased in the synthetic solution in all cases, with values in the 0.6–3.4 and 0.7–1.9 mg NH_3_-N∙cm^−2^∙d^−1^ interval for the 81.7 and 164.3 cm^2^ membrane surface areas, respectively. These results are close to those obtained by Fillingham, et al. [46], who reported a NH_3_-N mass flux of 0.76 mg·cm^−2^·d^−1^ at concentrations of 3280 mg TAN·L^−1^ for an ePTFE membrane in a suspended system, and higher than those obtained by other authors [7,20,27] for ePTFE or PDMS membranes in submerged systems.

### 3.2. Effect of Acid Flow Rate

The effect of the acid flow rate on NH_3_ capture effectiveness was evaluated for an intermediate membrane surface area (122.5 cm^2^) and synthetic N emitting solution concentration (6000 mg NH_3_-N·L^−1^), selected taking into account the average value of N concentration of a homogenized pig slurry from the community of Castilla y León (viz., 5.43 g·L^−1^) [47]. During the experiments, pH values of 0.5 ± 0.2 and 8.3 ± 0.1 were registered in the acidic and the synthetic solutions, respectively, and assays were carried out at room temperature (21.0 ± 2 °C). The initial NH_3_-N content in the synthetic solutions ranged from 5985 to 6240 mg.

The NH_3_-N mass removed from the synthetic N emitting solution and recovered by the acidic solution, and the N flux for each acid flow rate are presented in Table 2.

The percent ammonia removal from the synthetic solution ranged from 41 to 68%, for 0.8 and 2.1 L·h^−1^ flow rates, respectively.

**Figure 3:**
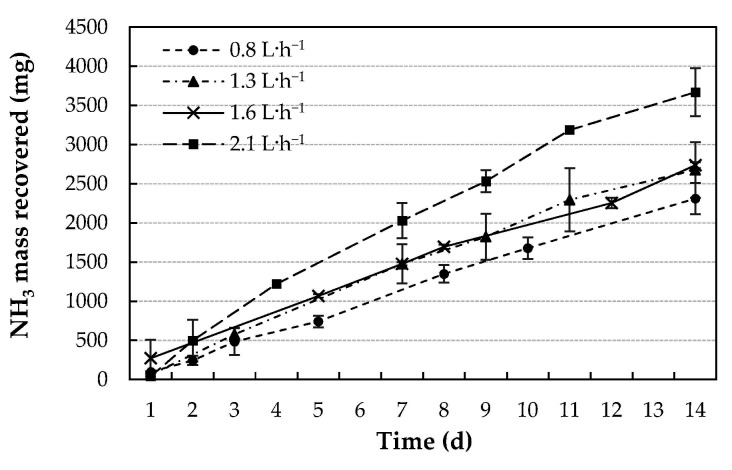
NH_3_ mass recovered in the acidic solution for 0.8, 1.3, 1.6, and 2.1 L·h^−1^ acid flow rates over a 14-day period with a 6000 mg NH_3_-N·L^−1^ concentration in the N emitting solution.

Significant differences in terms of the recovered NH_3_ mass were observed as a function of the flow rate, with the highest value (3669 ± 30 mg N) for the highest flow rate (2.1 L·h^−1^). In fact, the ammonia recovery was 37% higher for the highest flow rate than for the lowest one (0.8 L∙h^−1^). This is in good agreement with the results reported by Majd and Mukhtar [34], who found that increasing the flow rate of the receiving solution from 5.6 to 36 mL·min^−1^ (0.3–16.8 L∙h^−1^) led to an increase in the recovered NH_3_ mass of 30%. It should be clarified that the accumulation of NH_3_ in the acidic solution was linear in all cases (R^2^ > 0.98), as shown in Figure 3.

The percentage of NH_3_-N recovery was higher than in the first experiment, with values in the 88.9–92% range. Such values are substantially higher than those obtained by, for instance, Rothrock, et al. [27] (in the 67.7–76.2% range).

The ammonia capture rates per day ranged from 165 ± 14 to 262 ± 22 mg NH_3_-N·d^−1^ for the 0.8 and 2.1 L·h^−1^ flow rates, respectively. This result is consistent with the literature, in which it has been demonstrated that increasing the flux of the acidic solution improves ammonia capture [33,34,46], given that a faster acid flux removes NH_3_ molecules from the membrane faster and opens spaces for adjacent NH_3_ molecules to better diffuse through the membrane, reducing the effect of the boundary layer [48].

In relation to the N flux, significant differences were also observed, with *J* values between 1.35 and 2.14 mg NH_3_-N∙cm^−2^∙d^−1^ for the 0.8 and 2.1 L·h^−1^ flow rates, respectively. The increase in speed generates turbulences in the acidic solution, improving the reaction between ammonia and sulfuric acid, and reducing the thickness of the boundary layer [32]. For instance, an increase in the acidic solution flow from 0.83 to 1.25 L·h^−1^ was reported to increase the NH_3_-N mass flow from 2.1 to 2.5 mg NH_3_-N∙cm^−2^∙d^−1^ using a membrane with similar characteristics [49]. In contrast, Majd and Mukhtar [34] did not observe proportionality between the increase in the mass flow and the increase in the flow rate of the capture solution because the initial NH_3_ concentration in the corresponding liquid manure decreased over the experimental period. These authors determined mass flow rates between 0.66 and 0.77 g NH_3_-N∙cm^-−2^∙d^−1^ for flow rates of 5.6, 11, 23, and 36 mL·min^−1^ (i.e., 0.34–2.16 L·h^−1^).

### 3.3. Effect of Ammonia Capture Solution

Combining the parameters for which the best results were obtained in terms of NH_3_ capture in previous sections, the effect of the type of entrapment solution on NH_3_ capture was then evaluated. A surface area of 163.4 cm^2^ and a concentration of synthetic N emitting solution of 6000 mg NH_3_-N·L^−1^ were selected, using a constant flow of liquid inside the membrane of 2.1 L·h^−1^.

During the experiments, the pH of the capture and synthetic solutions was controlled. To control the different pHs of the capture solutions, a digital pH-meter with continuous reading was used. In the case of the pH of the synthetic solutions, the pH was measured on the samples taken. The pH of the acidic solution was kept < 2, that of the carbonated water solution was kept < 6.36, and that of the water solution was kept at pH < 9.2 (Table 3). The pH of the synthetic solutions was kept around pH 8 in all cases. The temperature (2 °C) was also maintained in all the traps so that the results could be compared.

The mass of NH_3_-N removed from the synthetic N emitting solution and recovered by the ammonia capture solutions and the N flux for each type of ammonia capture solution are summarized in Table 3.

The percentage of ammonia removal from the synthetic solution was 29, 12, and 33% in the circuits filled with 1N H_2_SO_4_, carbonated water, and water, respectively.

Significant differences were not observed in terms of the NH_3_ mass captured between the water and sulfuric acid traps, while significant differences were observed between the former two and the carbonated water traps. The highest value was attained for the water traps (1760 ± 134 mg NH_3_), followed by the sulfuric acid traps (1602 ± 73 mg NH_3_), and finally by the carbonated water traps (414 ± 36 mg NH_3_). However, the percentage of ammonia recovery using acid traps was the highest (88%), followed by water traps (81%), and carbonated water traps (49%).

The good performance of the sulfuric acid capture solution was expected due to the strong interaction between ammonium and the anion from the dissociation of the acid, leading to the formation of (NH_4_)_2_SO_4_. Other authors have also reported an optimal behavior of sulfuric acid in the ammonia capture process [41,50,51,52].

On the other hand, the results obtained when water was tested as the ammonia capture solution were unexpected. This could happen if not only NH₄OH is formed in the trap, but some of the captured ammonia is also retained in solution as NH_3_ (aq). Hence, the observed behavior may be tentatively explained by the high solubility of NH_3_ in water due to its polarity: NH_3_ forms hydrogen bonds with water molecules, which would be favored by the decrease in temperature [53,54,55]. Reducing the temperature of the NH_3_/NH_4_^+^ solution increases the solubility of ammonia in water and changes the dynamic equilibrium between the two species at more basic pHs [56].

In contrast, Damtie, et al. [41] observed a trend of slow absorption of NH_3_ since, once the pH reached the value of 9.2 and the NH_3_ concentration reached saturation, no NH_3_ transfer took place (NH_3_ could even migrate towards the feed side). Using water as an absorbent in capacitive membrane extraction systems (CapAmm), Zhang, et al. [52] reported NH_3_ recovery efficiencies of 35%, while efficiencies > 70% were achieved with non-volatile acids such as H_2_SO_4_ and H_3_PO_4_. A back diffusion of NH_3_ and an occupation of the pores of the membrane took place, deteriorating the NH_3_ recovery performance.

It is worth noting that the NH_3_ recovery efficiency reported herein when carbonated water was used as the absorber was 48.7%, very similar to that obtained by Zhang, et al. [52] with CapAmm (48.3%). The authors explained that the use of H_2_CO_3_ as an absorbent produced a backscattering of CO_2_ and NH_3_, as a result of the competitive occupation of the membrane pores, which led to a deterioration in the NH_3_ flow network.

Regarding the N flux, significant differences were also observed between the acid and water traps with respect to the carbonated water traps, with values of 1.4, 1.5, and 0.4 mg NH_3_-N∙cm^2^∙d^−1^, respectively. Since the NH_3_ mass captured in the acid and water traps was similar, the recovery by surface area was similar too.

Finally, it should be noted that the use of chilled water as an alternative ammonia capture solution would entail important savings. The main advantage would be that restricting the use of acids as ammonia removal solutions would prevent the handling of hazardous chemicals. Furthermore, according to the economic study carried out by Zhang, et al. [52], the use of chilled water would be more attractive than those involving strong acid adsorbents, given that acids require a high investment in chemical products and the final product obtained is cheap. Further, if water was used as a capture solution, the only operative cost would be energy consumption. In the case of using water cooled down to 2 °C as the capture solution, the possible re-emission should be considered if the temperature increases. However, if the system is operated in hermetic conditions, the ammonia cannot escape, so there would be no possibility of generating associated emissions. Regarding the use of the final product obtained, the use of water as a capture solution would generate a higher value final fertilizer product, NH_3·_H_2_O (5$·kg^−1^ N), than that obtained using an acidic solution, (NH_4_)_2_SO_4_ (0.5$·kg^−1^ N) [52]. In the case of ammonium bicarbonate, its main use would be as a fertilizer [57]. The demand for fertilizer products is expected to continue increasing and industrial uses of N grow even faster. Therefore, the price of this type of nitrogenous solution is expected to increase at least until a 2030 horizon, according to Heffer and Prud’homme [58]. In the case of ammonia solutions, they also have a potential use for the control of NO_x_ emissions, in the dyeing, wood, and leather industries, or in detergents.

## 4. Conclusions

Suspended ePTFE gas-permeable membrane technology was effective for the recovery of gaseous NH_3_ using a closed loop system, with percentages of NH_3_-N recovery of up to 92%. The different membrane surface areas, NH_3_-N concentrations in the emission source, flow rates of the acidic solution, and types of NH_3_ capture solutions resulted in statistically significant differences in terms of NH_3_ capture in the gas phase. While the increase in membrane surface area led to a proportional increase in the recovered NH_3_ mass at 3000 and 6000 mg NH_3_-N·L^−1^, at the highest concentration (12,000 mg NH_3_-N·L^−1^) this increase was much smaller, pointing to the existence of other limiting factors, such as the flow rate. In this regard, a 37% increase in NH_3_ recovery was attained by increasing the acid flow rate from 0.8 to 2.1 L·h^−1^. In the optimized conditions, chilled water was utilized as a NH_3_ capture solution, finding high NH_3_ recovery rates, comparable to those obtained using a sulfuric acid. This opens the possibility of using chilled water to capture NH_3_ from animal housing instead of acidic solutions, with associated savings. The presented results suggest that suspended GPM systems hold great promise, but evidence the importance of fine-tuning system parameters in order to optimize NH_3_ capture.

## Figures and Tables

**Table 1:** Initial NH_3_ mass in the synthetic solution (Initial N-NH_3_), NH_3_ mass removed from the liquid (Removed N-NH_3_), NH_3_ mass recovered in the acidic solution (Recovered N-NH_3_), and N flux as a function of the NH_3_-N concentration in the N emitting solution for two membrane surface areas (81.7 and 163.4 cm^2^).

Membrane Surface Area (cm^2^)	Theoretical NH_3_-N Concentration (mg·L^−1^)	Initial NH_3_-N (mg N)	Removed NH_3_-N (mg N)	Recovered NH_3_-N (mg N)	N Flux (mg·cm^−2^·d^−1^)
81.7	3000	2743 ± 68	840 ± 103	663 ± 93 e	0.6 ± 0.1 d
	6000	5929 ± 454	1748 ± 366	1545± 62 d	1.4 ± 0.1 c
	12,000	13,153 ± 93	5713 ± 499	3925 ± 101 b	3.4 ± 0.1 a
163.4	3000	3102 ± 74	1655 ± 104	1609 ± 10 d	0.7 ± 0.0 d
	6000	6167 ± 689	3106 ± 352	2993 ± 54 c	1.3 ± 0.0 c
	12,000	11,744 ± 85	4954 ± 1015	4366 ± 14 a	1.9 ± 0.0 b

Values followed by the same letter are not significantly different at *p* ≤ 0.05 according to Tukey’s HSD test. All values are expressed as mean ± s.d. of *n* = 3.

**Table 2:** Initial NH_3_ mass in the synthetic solution (Initial N-NH_3_), NH_3_ mass removed from the liquid (Removed N-NH_3_), NH_3_ mass recovered in the acidic solution (Recovered N-NH_3_) and N flux as a function of the acid flow rate.

Flow Rate (L∙h^−1^)	Initial N-NH_3_ (mg N)	Removed N-NH_3_ (mg N)	Recovered N-NH_3_ (mg N)	N Flux (mg N∙cm^−2^∙d^−1^)
0.8	6240 ± 107	2583 ± 324	2311 ± 200 c	1.35 ± 0.12 c
1.3	6039 ± 9	2935 ± 431	2676 ± 356 bc	1.56 ± 0.21 bc
1.6	5985 ± 250	2974 ± 86	2737 ± 40 b	1.60 ± 0.02 b
2.1	6108 ± 517	4128 ± 470	3669 ± 305 a	2.14 ± 0.18 a

Values followed by the same letter are not significantly different at *p* ≤ 0.05 according to Tukey’s HSD test. All values are expressed as mean ± s.d. of *n* = 3.

**Table 3:** Initial NH_3_ mass in the synthetic solution (Initial N-NH_3_), NH_3_ mass removed from the liquid (Removed N-NH_3_), NH_3_ mass recovered in the acidic solution (Recovered N-NH_3_), and N flux as a function of the ammonia capture solution.

Stripping Solution	Initial pH	Final pH	Initial N-NH_3_ (mg N)	Removed N-NH_3_ (mg N)	Recovered N-NH_3_ (mg N)	N Flux (mg N∙cm^−2^∙d^−1^)
1N H_2_SO_4_	0.3 ± 0.1	0.5 ± 0.2	6230 ± 90	1777 ± 166	1602 ± 73 a	1.4 ± 0.1 a
Carbonated water	4.4 ± 0.1	6.3 ± 0.1	6285 ± 122	828 ± 135	414 ± 36 b	0.4 ± 0.0 b
Water	6.5 ± 0.4	8.1 ± 0.1	6380 ± 335	2128 ± 216	1760 ± 134 a	1.5 ± 0.1 a

Values followed by the same letter are not significantly different at *p* ≤ 0.05 according to Tukey’s HSD test. All values are expressed as mean ± s.d. of *n* = 3.

## Data Availability

The data presented in this study are available on request from the corresponding author. The data are not publicly available due to their relevance as part of an ongoing Ph.D. Thesis.
